# Retinal degeneration in spinocerebellar ataxia type 7: an overview of the current knowledge

**DOI:** 10.5935/0004-2749.2024-0248

**Published:** 2025-02-13

**Authors:** Bruna Ferraço Marianelli, Flávio Moura Rezende Filho, Mariana Vallim Salles, José Luiz Pedroso, Orlando Graziani P. Barsottini, Juliana Maria Ferraz Sallum

**Affiliations:** 1 Division of Retina and Vitreous, Department of Ophthalmology, Universidade Federal de São Paulo, São Paulo, SP, Brazil.; 2 Division of General Neurology and Ataxia Unit, Department of Neurology and Neurosurgery, Universidade Federal de São Paulo, São Paulo, SP, Brazil.

**Keywords:** Cerebellar ataxia, Cone-rod dystrophies, Spinocerebellar ataxias, Retinal dystrophies, Retinal degeneration, Genetic therapy

## Abstract

Spinocerebellar ataxia type 7 is a form of spinocerebellar ataxia, which is a clinically and genetically heterogeneous group of rare inherited neurodegenerative disorders. Among the spinocerebellar ataxias, the association between cerebellar ataxia and cone-rod retinal dystrophy is a strong indicator of spinocerebellar ataxia type 7. Spinocerebellar ataxia type 7 cone-rod dystrophy is a progressive, disabling, and incurable form of hereditary retinopathy. However, the field of genetics has markedly progressed in the last decades, which resulted in improved understanding of multiple aspects of spinocerebellar ataxia type 7 retinal degeneration and the emergence of new modalities of genetic therapies for other types of retinal dystrophies. This study aimed to evaluate the current knowledge on spinocerebellar ataxia type 7 retinal degeneration, including genetics and molecular mechanisms as well as their implications in pathogenesis, clinical manifestations, and potential therapeutic strategies.

## INTRODUCTION

Spinocerebellar ataxia type 7 (SCA7) is a form of spinocerebellar ataxia (SCA), which is defined as a heterogeneous group of inherited neurodegenerative disorders^(1)^. The most common SCAs share the same core genetic mechanism, an expanded cytosine-adenine-guanine (CAG) repeat in the causative gene (ataxin gene *[ATXN]),* and neurological features due to degeneration of the cerebellum, brainstem, and spinal cord, resulting in ataxia, dysphagia, dysarthria, pyramidal and extrapyramidal signs, peripheral neuropathy, and ophthalmoparesis^(2)^. In SCA7, the expansion occurs on the *ATXN7* gene, with autosomal dominant transmission. SCAs may also result from point mutations, DNA rearrangements, and expansions of the noncoding repeats of other genes.

The big family of SCAs encompasses more than 50 subtypes of the disease, the most common being 1, 2, 3, 6, 7, 10, and 17. Despite having many subtypes, SCA is a rare condition; the estimated prevalence of SCA worldwide is 3:100,000, with SCA7 alone having a frequency of 1:500,000^(3)^. SCA7 is more prevalent in countries with a founder effect, such as Sweden, Norway, Denmark, Finland, South Africa, and Mexico^(4)^. In the Brazilian population, SCA7 is the third most common subtype next to SCA3 (also know as Machado-Joseph disease) and SCA2^(5)^.

Cerebellar ataxia is the main feature of SCA and may manifest in isolation, such as in subtypes 5, 6, and 11, or in combination with additional neurological signs (peripheral neuropathy, pyramidal signs, dystonia, myoclonus, parkinsonism, and others), such as in subtypes 1, 2, and 3^(6)^. Among the SCAs, the association between cerebellar ataxia and cone-rod retinal dystrophy is a strong indicator of SCA7 and thus constitutes a key feature of the latter. The presence of retinal dystrophy in a patient with SCA7 facilitates differential diagnosis with other SCA subtypes, although retinal involvement has been reported in SCA1 and rarely in SCA2. Cone-rod dystrophy is an extremely important trait of the SCA7 phenotype, which may lead to severe visual impairment and greatly affect the lives of the patients, particularly in the final stages of the disease^(7)^.

SCA7 cone-rod dystrophy is a progressive, disabling, and incurable form of hereditary retinopathy. However, the field of genetics has substantially progressed in the last decades, resulting in improved understanding of the multiple aspects of SCA7 retinal degeneration and the emergence of new modalities of genetic therapies for other types of retinal dystrophies. SCA7 is an interesting model for the study of retinal involvement due to the presence of trinucleotide expansion diseases, a group of neuropsychiatric disorders caused by abnormal expansions of repetitive trinucleotide sequences, such as CAG in SCAs or cytosine-thymine-guanine in myoto-nic dystrophy. This study aimed to review the current knowledge on SCA7 retinal degeneration, including genetics and molecular mechanisms as well as their implications in pathogenesis, clinical manifestations, and potential therapeutic strategies.

### Genetics and molecular mechanisms

SCA7 is an autosomal dominant neurodegenerative disorder^(8)^. The genetic mechanism responsible for SCA7 is the expansion of the CAG trinucleotide that codes the amino acid glutamine in the ataxin7 *(ATXN7)* gene, which is located in the short arm of chromosome 3 (locus 3p14.1)^(9)^. While healthy *ATXN7* alleles contain 4 to 36 CAG repetitions (more typically below 17), pathogenic alleles contain 37 to 460^(10)^.

The anticipation phenomenon occurs under some genetic conditions; it is defined as a more severe and/or earlier presentation of the disease as it is passed from one generation to another^(11)^. This phenomenon is a remarkable feature in SCA7, which means that there is a strong possibility of expansion of the CAG repetition length through consecutive generations^(12)^. The magnitude of CAG expansion affects the age of presentation, disease duration, and clinical severity, with larger expansions associated with earlier onset of signs and symptoms, more severe and accelerated clinical course, and lower life expectancy^(13)^.

The *ATXN7* gene encodes the ataxin7 protein, a core component of SPT3 acetyltransferase (SAGA) complexes, that is involved in chromatin remodeling processes^(14)^. To enable transcription, chromatin must undergo some structural changes to facilitate the access of transcription factors to DNA^(15)^. SAGA complexes are involved in histone acetylation, which helps promoters (specific DNA sequences recognized by transcriptional factors that signal the transcription initiation) access transcription factors by unzipping chromatin^(16)^. Furthermore, ataxin7 is a component of the USP22 deubiquitinating complex, which supports the initiation and elongation of transcription^(17)^.

The mutant ataxin7 contributes to retinal degeneration through several mechanisms: accumulation of mutant protein and consequent toxicity to tissues, induced transcriptional perturbations on photoreceptor genes, and possible involvement with caspase-7 proteolysis processes. Those processes will be discussed in the next paragraphs.

SCA7 is a representative of polyglutamine (polyQ) disorders, which are caused by expanded CAG repeats encoding a long polyQ tract in the respective proteins^(18)^. The mutant ataxin7 protein harbors a polyQ expansion, which confers toxic properties to the molecule and interfere with its final shape, leading to misfolding. As a result of the mutation and misfolding, a prominent intracellular accumulation of the mutant ataxin7 occurs in multiple human tissues and organs, including the cerebellum, brain, retina, and other peripheral tissues (intestine, thyroid gland, testis, smooth and cardiac muscle fibers, and others)^(19)^. Despite its ubiquitous expression in multiple organs, the cerebellum and retinal photoreceptors are the ones most vulnerable to its effects. To date, the cause of this phenomenon remains unclear. Progressively, protein accumulation generates mutant ataxin7 aggregates, which are identified as nuclear inclusions (NIs) via immunohistochemistry and are similar to amyloid-like aggregates^(20)^. Postmortem analyses revealed that the NI concentration is more accentuated in more vulnerable targets, such as retinal photoreceptors and Purkinje cells, than in other neurons^(21)^. The specific role of NIs in the pathogenesis of polyQ disorders has not yet been sufficiently investigated ^(22)^.

The proteolysis process seems to play a pivotal role in mutant ataxin7 accumulation and toxicity. It has been demonstrated that caspase-7 can produce mutant ataxin7 fragments by cleavage, which are more cytotoxic than the full-length protein^(23)^. A study involving a SCA7 mice model reported that a mutant ataxin7 fragment was one of the major components of NIs^(24)^. In addition, transgenic mice with mutations at the caspase-7 cleavage site exhibited milder clinical presentations than mice without this mutation, with reduced neurodegeneration, better visual and motor performances, and extended survival, indicating the importance of proteolysis in the pathogenesis of SCA7 as well as the protector role of mutations downregulating the caspase-7 cleavage process^(25)^.

Transcriptional changes constitute another relevant mechanism implicated in the SCA7 pathogenesis. Abnormal transcription has been demonstrated to promote downregulation of photoreceptor-specific and cerebellar neuronal genes essential for the function of dendrites and myelin sheath^(26)^. The cellular and mouse models of SCA7 indicate that dysfunctions in SAGA complex acetylation and deubiquitination activities disrupt chromatin organization, leading to changes in the expressions of some genes^(27)^. The effects of transcriptional alterations on photoreceptor genes have been explored in numerous studies. The cone-rod homeobox protein (CRX), a major transcriptional factor of photoreceptor genes, was first implicated in SCA7 pathogenesis^(28)^. CRX was found to be downregulated in SCA7. Furthermore, dysfunction was identified on other transcriptional programs involved in the maintenance of mature pho-toreceptors, such as neural retina leucine zipper protein (NRL) and nuclear receptor subfamily 2, group E, member 3 (NR2E3) were both found to be downregulated, whereas OPTX2, STAT3, and HES5, which inhibit the differentiation of precursor neurons into mature photo-receptors, were reactivated^(27)^.

### Ocular clinical manifestations

Visual system involvement is a key feature in the natural history of SCA7 and is caused by retinal degenera-tion^(29)^. Ophthalmological manifestations are present in 70%-100% of patients with SCA7. More typically, visual complaints occur after neurological manifestations, but these manifestations can be the first symptoms of SCA7, particularly in patients with greater CAG repeats, often exhibiting infantile (symptom onset shortly after birth and death in the first or second year) and juvenile forms (onset during childhood or adolescence). In SCA7, retinal degeneration has a progressive clinical course. In the initial stages, there is a predominant involvement of cone photoreceptors, concentrated in the macular region, which is consistent with a cone dystrophy phenotype. In this stage, ocular manifestations include dyschromatopsia, photophobia, and central vision loss. In the final stages, the disease progresses to the peripheral retina and affects the rod photoreceptor, resulting in a cone-rod dystrophy phenotype. At this point, diffuse vision loss occurs and ultimately progresses to complete blindness^(30^,^31^,^32)^.

**Figure 1:**
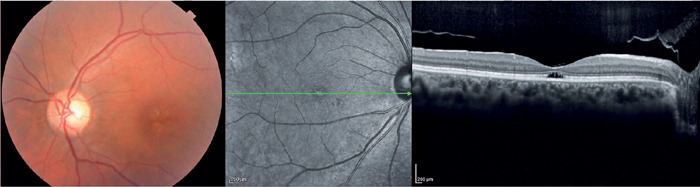
Color fundus photography shows macular pigmentation abnormalities with a granular appearance. OCT shows loss of the ellipsoid zone in foveal topography and outer retina hole.

**Figure 2:**
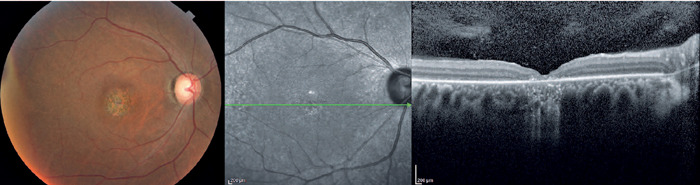
Color fundus photography and OCT show macular atrophy

**Figure 3:**
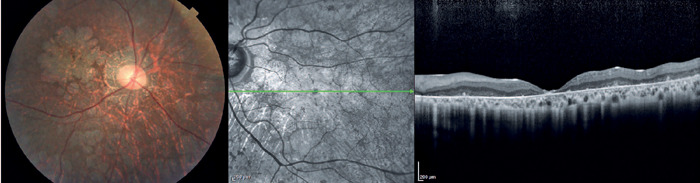
Color fundus photography and OCT show diffuse nummulaire confluent atrophic lesions, including atrophy at the macula, around the optic disc, and contiguous to vascular arcades. The associated findings include optic disc pallor and prominent vascular thinning.

In a previous study, we proposed a classification for the successive stages of SCA7 retinal degeneration^(33)^. A total of 20 consecutive patients (40 eyes) with mole-cularly confirmed SCA7 diagnosis were evaluated via slit-lamp and fundus examination, color fundus photography, and spectral domain optical coherence tomography. Furthermore, a retina specialist conducted a qualitative analysis as well as described in detail all the pathological features and developed a graduation system for SCA7 retinal degeneration (Table 1). The first signs of retinal degeneration are the absence of normal foveal reflex and abnormalities in macular pigmentation, such as granular appearance, similar to the early findings of cone dystrophies (Figure 1)^(33)^. In this stage, OCT shows abnormalities in outer retinal layers, including loss of the ellipsoid zone and disruption of the inner-outer segment junction of the photoreceptors and/ or subfoveal cavitation (outer retina hole) (Figure 1)^(34)^. Thereafter, these macular abnormalities progress to different degrees of macular atrophy, including geographic atrophy (Figure 2)^(35)^. In the final stages, a generalized dystrophy occurs, with diffuse nummulaire confluent atrophic lesions, including atrophy at the macula, around the optic disc, and contiguous to vascular arcades^(33)^. The associated findings include optic disc pallor and prominent vascular thinning (Figure 3). Interestingly, the present study demonstrated a correlation between the stage of retinal degeneration and the severity of neurological disease, accessed by the SARA and ICARS scores^(33)^.

**Table 1:** Clinical features of retinal degeneration in spinocerebellar ataxia 7

SCA7 retinal degeneration stage	Fundus exam/color fundus photographs	Optical coherence tomography
**Stage 0**	Normal foveal reflex No macular abnormalities are identified	Normal foveal anatomy No macular abnormalities are identified
**Stage 1**	Abnormal foveal reflex Abnormalities in macular pigmentation (granular appearance)	Abnormalities in the outer retinal layers, including loss of the ellipsoid zone and disruption of the inner-outer segment junction of the photoreceptors and/or subfoveal cavitation (outer retina hole)
**Stage 2**	Pigmentary abnormalities and different degrees of macular atrophy	Different degrees of macular atrophy
**Stage 3**	Generalized dystrophy with nummular confluent atrophic lesions at the macula, around the optic disc, and contiguous to vascular arcades Optic disc pallor and prominent vascular thinning	Diffuse outer retina and retinal pigment epithelium atrophy on macular topography

Aside from retinal features, patients with SCA7 also exhibit eye motility abnormalities, such as ptosis, slow saccades, ophthalmoplegia, and nystagmus. Oculomotor abnormalities occur early in the course of SCA7 and are correlated with disease stage, duration, and severity^(36)^. In the final stages, eye motility anomalies, head involuntary movements, and visual acuity tend to worsen, making clinical evaluation, particularly the acquisition of OCT images, and other ophthalmic complementary examinations challenging.

It is important for ophthalmologists to recognize SCA7 and its ocular manifestations as they are crucial in achieving an accurate diagnosis. Commercially available genetic tests for retinal dystrophies usually do not cover DNA repeat expansion diseases. Thus, a good ophthalmic evaluation, particularly for patients with ocular presentation associated with neurological signs and symptoms, will lead to an assertive clinical suspicion of this unusual diagnosis. Previous knowledge of this condition will guide ophthalmologist in deciding on specific molecular tests to detect ATXN7 expansions. SCA7 cannot be detected by sequence-based multigene panels, exome sequencing, or genome sequencing. Thus, “targeted analysis for CAG trinucleotide expansions on the SCA7 gene” is recommended.

### Potential therapeutic strategies

At present, there are no therapeutic strategies for preventing visual loss or improving visual function in patients with SCA7. Neurological disorders are also untreatable. Nevertheless, some symptoms can be relieved by physical neurorehabilitation and exercise^(37)^. In parallel with nonpharmacological intervention, different lines of research are aimed at finding potential therapeutic strategies to prevent SCA7 and reverse its effects.

A very promising strategy for SCA7 and other polyQ neurodegenerative disorders that has been used in recent years is the use of gene silencing molecules to inhibit the expression of toxic proteins, which are products of mutated genes^(38)^. This treatment modality, also referred to as gene suppression therapy, includes different approaches, such as antisense oligonucleotides (ASOs) and RNA interference-based therapy (RNAi). These therapies have the potential to modify the disease course and can be applied while photoreceptor cells are still present and viable, even after the occurrence of initial damages.

ASOs consist of synthetic single strands of nucleotides arranged in a complementary sequence and designed to specifically bind to a target RNA. They combine with RNA, producing a DNA-RNA heteroduplex, which serves as substrate for RNase H degradation^(39)^. The final effects of ASO on the target RNA includes its degradation, prevention of its translation, and modification of its processing. An example of ASO applicability in humans is Spinraza® (Nusinersena, Biogen). Spiranza® is a drug whose mechanism is ASO, approved for use in patients with autosomal recessive spinal muscular atrophy (SMA), which is the leading genetic cause of infant mortality. SMA is caused by homozygous mutation or deletion of the SMN1 gene, responsible for coding the survival motor neuron (SMN) protein, which is essential for maintaining a-motor neurons. Neural degeneration in SMA causes progressive motor dysfunction, leading to death by respiratory insufficiency. Interestingly, humans carry a second copy of the SNM gene, namely, SMN2, which produces an unstable and partially functional protein under normal conditions due to constitutional splicing issues caused by mutation in exon 7. Spinraza^®^ is delivered via intrathecal injection and inhibits the inclusion of exon 7 on the translational products of SMN2 gene, thereby improving splicing and generating a stable and fully functional SMN protein^(40)^.

In 2018, Niu C et al. published the results of a pioneer study of ASOs targeting mutant ataxin 7. Delivered via intravitreal injections in a mice model, two types of ASO were tested: one directed against ataxin 7 RNA and the other against CAG repeat-containing RNA. The ASO targeting mutant ataxin 7 RNA was found to yield more favorable outcomes. Mice treated with ASO exhibited substantial reductions in ataxin 7 expression and protein aggregation, improvement in vision loss as well as cone and rod photoreceptor function documented via electroretinogram (ERG) tests, and amelioration of retinal histopathology, with less thinning in photoreceptor retinal layers^(41)^.

RNAi molecules play a role similar to that of ASOs. They consist of a double-stranded RNA intracellularly processed by Dicer (RNase III family ribonuclease), which transforms it into a small interfering RNA. Subsequently, the small interfering RNA is incorporated into the RNA-induced silencing complex, which binds to the targeted mRNA and destroys it. The ultimate result is the inhibited translation of targeted RNAm. In 2014, Davidson et al. published a study on RNAi therapy for SCA7^(42)^. Inspired by the results of previous studies on RNAi suppression in polyQ disease models (Huntington’s disease, SCA1, and SCA3), they tested the effects of RNAi in a BAC-Prp-SCA7-92Q mouse that expressed human mutant ataxin 7 cDNA in the central nervous system and retina. Unfortunately, the mouse did not exhibit the typical SCA7 retinal phenotype on ERG recordings, fundoscopy, and histological evaluation. Meanwhile, the mutant gene expression in the retina was confirmed via RT-qPCR and Western blot. With subretinal injections of AAV1/2, they reduced the transcript levels to 60%-70%, which resulted in a 45% reduction in the levels of mutant human ataxin 7 protein. Extrapolating this setting for human treatment, the study group estimated a 50% reduction in both normal and wild-type alleles, leaving 25% normal ataxin 7 protein. In addition, no safety issues were raised as histological analyses and ERG records on the treated retinas did not reveal any differences from the controls, indicating the tolerability of the therapy.

Aside from genetic therapies, other alternatives for treating SCA7 that target neurological and/or retinal diseases are being investigated. Interferon beta, which is already utilized to treat multiple sclerosis, may be useful in delaying motor symptoms in SCA7. Interferon beta stimulates mutant ataxin7 clearance by inducing nuclear PML-clastosomes. In a preclinical trial, Sittler et al. have demonstrated a decrease in mutant ataxin7 aggregation and improvement in motor functions following successive intraperitoneal injection of interferon beta in a SCA7 mouse model^(43)^. Another potential strategy, more specifically designed against neurological features, is the prevention of cerebellum Purkinje cell degeneration in SCA7. An excess in glutamate is known to potentially increase cell vulnerability to excitotoxicity-induced degeneration. Meanwhile, ceftriaxone promotes glutamate clearance by stimulating GLT-1 expression^(38)^. In fact, studies involving SCA28 mice administered ceftriaxone reported prevention of ataxia onset on presymptomatic stages and inhibited progression on postsymptomatic stages^(44)^. The therapeutic methods that are being considered in other cone-rod dystrophies and retinal degenerative disease where photoreceptor cells are lost have potential applications in SCA7 retinal degeneration, including stem cell-derived photoreceptor cell replacement, electronic retinal implants, and optogenetics^(45)^.

SCA7 is a rare disease characterized by progressive ataxia and visual loss caused by retinal degeneration with cone-rod dystrophy phenotype. It is crucial for ophthalmologists to recognize this entity and its ocular features as they help achieve an accurate diagnosis, which rely on specific genetic tests. Gene suppression therapies, such as ASOs and RNAi, have the potential to modify the disease course and to be applied while pho-toreceptor cells are still present and viable, indicating the importance of early diagnosis.
